# Standardized femoral artery cannulation and ischemia-prevention protocol for minimally invasive cardiac surgery

**DOI:** 10.1016/j.xjtc.2026.102485

**Published:** 2026-06-15

**Authors:** Shota Inoue, Kohei Nagashima, Mitsuru Yokoyama, Nariaki Nagaoka, Hiroshi Nakanaga, Chikara Ueki, Atsuhiko Sato, Shigefumi Matsuyama, Minoru Tabata

**Affiliations:** a Department of Cardiovascular Surgery, Toranomon Hospital, Tokyo, Japan; b Department of Clinical Engineering, Toranomon Hospital, Tokyo, Japan; c Department of Cardiovascular Surgery, Juntendo University School of Medicine, Tokyo, Japan

**Keywords:** minimally invasive cardiac surgery, femoral artery cannulation, lower-limb ischemia, regional oxygen saturation, distal perfusion

## Abstract

**Objectives:**

In minimally invasive cardiac surgery, femoral artery cannulation is widely used; however, it may cause lower-limb ischemia. We developed a standardized patient-selection strategy, a femoral artery–cannulation technique, and a structured ischemia-prevention protocol and evaluated their reproducibility and clinical effectiveness in preventing limb ischemia.

**Methods:**

We retrospectively analyzed 505 patients who underwent totally endoscopic minimally invasive cardiac surgery with femoral artery cannulation between June 2019 and July 2024. The arterial cannula was inserted with the proximal side hole directed dorsally and secured at a shallow depth to facilitate distal perfusion. Regional oxygen saturation of both lower limbs was continuously monitored. A >50% decrease in regional oxygen saturation triggered a stepwise protocol: increased cardiopulmonary bypass flow, topical papaverine hydrochloride solution, and, if unresolved, distal perfusion via a 6-Fr sheath. The incidences of clinical limb ischemia (need for distal perfusion or compartment syndrome) and a >50% decrease in regional oxygen saturation were evaluated, and risk factors for regional oxygen saturation decrease were identified.

**Results:**

No patients required distal perfusion or developed postoperative compartment syndrome. A >50% decrease in regional oxygen saturation occurred in 28 patients (5.5%), all of whom recovered without distal perfusion. Multivariate analysis identified smaller femoral artery diameter and low ejection fraction as independent risk factors for regional oxygen saturation decrease.

**Conclusions:**

A standardized patient-selection strategy combined with a femoral artery–cannulation technique and a structured ischemia-prevention protocol effectively prevented clinically significant limb ischemia without the need for distal perfusion in this cohort.

**Figure:**
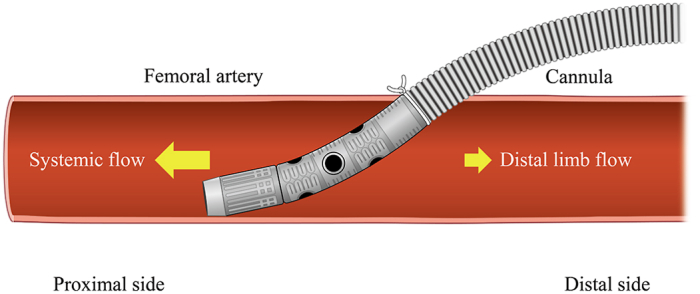
Standardized femoral artery–cannulation technique ensuring reliable distal flow.

**Central Message**

A standardized femoral artery–cannulation technique combined with a structured ischemia-prevention protocol enables safe and reproducible perfusion during minimally invasive cardiac surgery.

**Perspective**

This study introduces a standardized femoral artery–cannulation technique combined with a structured ischemia-prevention protocol for minimally invasive cardiac surgery. By securing distal flow and reducing limb ischemia risk without increasing procedural complexity, this approach provides a reproducible and practical framework for safe femoral perfusion and may help optimize perfusion management.

In minimally invasive cardiac surgery (MICS), cardiopulmonary bypass (CPB) is commonly established through cannulation of the femoral artery (FA) and femoral vein (FV). Although this approach is widely used, femoral arterial perfusion carries a risk of lower-limb ischemia, which remains a clinically relevant complication.

Near-infrared spectroscopy (NIRS) has been validated as a reliable, noninvasive tool for monitoring regional oxygen saturation (rSO_2_) in the lower extremities. The reported incidence of significant rSO_2_ decrease during MICS varies among institutions, ranging from 4.3% to 27.3%.^1^, ^2^, ^3^ This wide variation suggests that technical factors, including the details of FA cannulation, may play a major role in determining distal perfusion (DP).

However, standardized approaches to patient selection, FA cannulation, and ischemia prevention are not well established, and only few studies have evaluated structured strategies designed to maintain distal limb perfusion during MICS. As a result, optimal, reproducible strategies for minimizing rSO_2_ decline and preventing lower-limb ischemia remain unclear. Therefore, this study aimed to evaluate the effectiveness of a standardized patient-selection strategy combined with a FA-cannulation technique and a structured ischemia-prevention protocol in preventing lower limb ischemia during MICS, using continuous intraoperative rSO_2_ monitoring.

## Methods

### Study Population

Between June 2019 and July 2024, 576 consecutive patients underwent totally endoscopic MICS at our institution. Among them, 70 patients who underwent axillary artery cannulation and 1 patient who underwent central artery cannulation were excluded. The remaining 505 patients, all of whom underwent FA cannulation using the standardized method and structured ischemia-prevention protocol, comprised the final study cohort.

### Patient Selection

**Figure E1:**
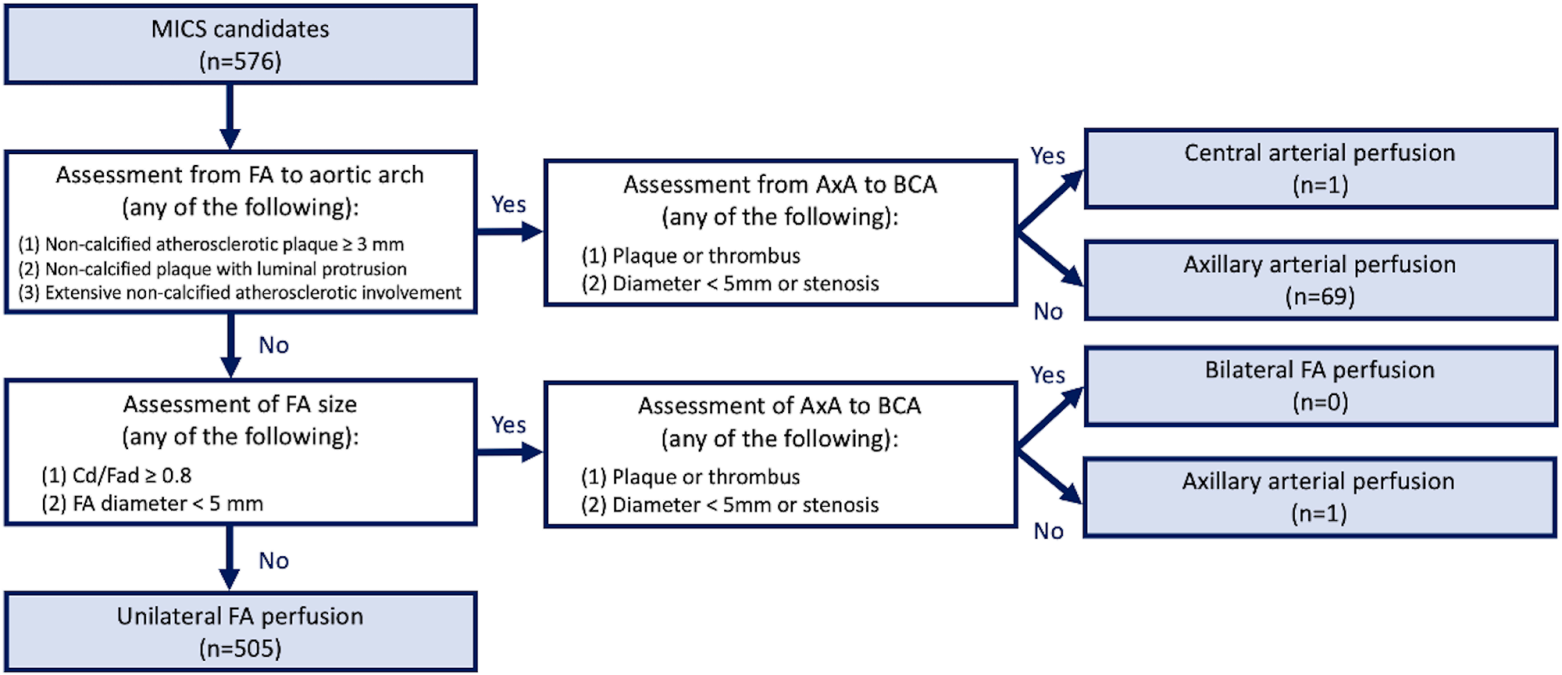
Computed tomography–based standardized preoperative screening strategy for cannulation site selection in minimally invasive cardiac surgery. *MICS*, Minimally invasive cardiac surgery; *FA*, femoral artery; *AxA*, axillary artery; *BCA*, brachiocephalic artery; *Cd/Fad*, cannula-to-femoral artery diameter ratio.

Patient selection was performed according to a standardized preoperative screening strategy (Figure E1). Specifically, all candidates for MICS underwent contrast-enhanced computed tomography (CT) to assess the arterial pathway from the femoral access site to the aortic arch. FA cannulation was avoided when any of the following findings were present: (1) markedly thick noncalcified atherosclerotic plaque (reference value ≥3 mm), (2) noncalcified plaque with luminal protrusion, or (3) extensive noncalcified atherosclerotic involvement. These criteria were applied collectively to exclude patients with significant noncalcified atherosclerotic burden.

Local vessel conditions at the planned cannulation site were also assessed. FA cannulation was avoided when the FA diameter was considered insufficient for the required cannula size. Specifically, cannula size was determined according to body surface area (Table E1). A cutoff value for the cannula-to-femoral artery diameter ratio (Cd/Fad) of 0.8 was applied; when this threshold was exceeded, the use of a cannula one size smaller than that recommended by body surface area was permitted. If this size adjustment was not feasible, FA cannulation was considered inappropriate. In addition, FA cannulation was uniformly avoided when the FA diameter was <5 mm. Although alternative strategies using a prosthetic side graft have been reported for small-caliber arteries, this approach was not adopted at our institution. Bilateral FA cannulation, which allows arterial inflow from 2 access sites, represents another alternative strategy and is considered an option for small-caliber arteries at our institution. However, no patients in the present series underwent bilateral FA cannulation. Among the excluded patients, 70 underwent axillary artery cannulation, of whom 69 had significant atherosclerotic disease of the aorta and 1 had bilaterally small FA. One patient underwent central cannulation because of both atherosclerosis and small axillary arteries. No patients in the present series underwent combined axillary and FA cannulation.

### Femoral Cannulation Techniques

All procedures were performed with the patient under general anesthesia via a totally endoscopic right minithoracotomy. CPB was established with FA perfusion and FV drainage, supplemented by internal jugular drainage in many cases. A centrifugal pump was used to maintain systemic flow at 2.2 to 2.5 L/min/m^2^, and arterial line pressure was continuously monitored and maintained below 300 mm Hg. Core temperature was maintained at 34 °C.

**Figure 1:**
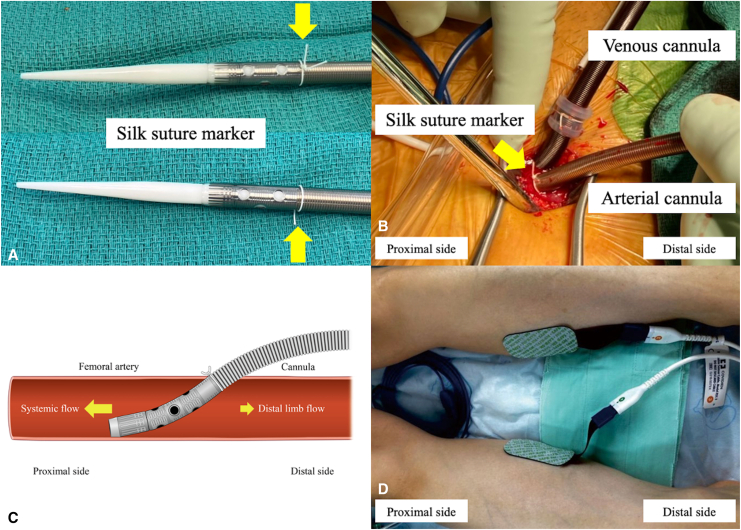
Technique of femoral artery cannulation and intraoperative monitoring of regional oxygen saturation. A, Marking with 3-0 silk suture on the intended cannulation line. B, Cannula insertion using the Seldinger technique, with dorsal orientation of the proximal side hole and advancement until the marker knot reached the arterial surface. C, Positioning of the cannula with the proximal side hole directed toward the distal femoral artery to promote antegrade limb perfusion. D, Placement of near-infrared spectroscopy sensors bilaterally on the gastrocnemius muscles for continuous monitoring of regional oxygen saturation.

For femoral cannulation, a 2- to 3-cm oblique incision was made in the groin crease to expose the anterior surfaces of the FA and FV, without circumferential dissection or vessel taping. Cannulation was performed in the sequence of vein first, followed by artery. In all patients, Bio-Medicus NextGen (Medtronic) cannulas were used for arterial cannulation; these reinforced-tip cannulas have 6 side holes. Before cannulation, a 3-0 silk suture was tied around the cannula along the planned insertion line, with the knot aligned coaxially with the proximal side port, serving as a marker. This marker therefore functioned both as a depth indicator to prevent excessive insertion and as a rotational guide to ensure correct side-port orientation (Figure 1, *A*).

A longitudinal purse-string suture with 5-0 polypropylene was placed along the axis of the FA. The arterial cannula was inserted using the Seldinger technique under transesophageal echocardiographic guidance, confirming wire position in the true lumen of the descending aorta. Using the silk marker as a reference, the cannula was inserted so that the knot was positioned at the arterial surface, thereby placing the most proximal side port approximately 4 to 5 mm within the arterial lumen. By aligning the knot ventrally, the dorsal proximal side port was oriented caudally toward the distal FA (Figure 1, *B* and *C*). The cannula was secured at 3 points to prevent dislodgement: (1) to the tourniquet of the purse-string suture, (2) to the skin near the incision, and (3) to the surgical drape.

### Intraoperative Limb Monitoring and Ischemia-Prevention Protocol

Limb perfusion was continuously assessed using an INVOS NIRS monitor (Medtronic). Sensors were bilaterally applied to the gastrocnemius muscles, which are muscle-rich regions considered important for evaluating the risk of compartment syndrome (Figure 1, *D*). Baseline rSO_2_ values were defined after the induction of anesthesia and before surgical incision.

**Figure 2:**
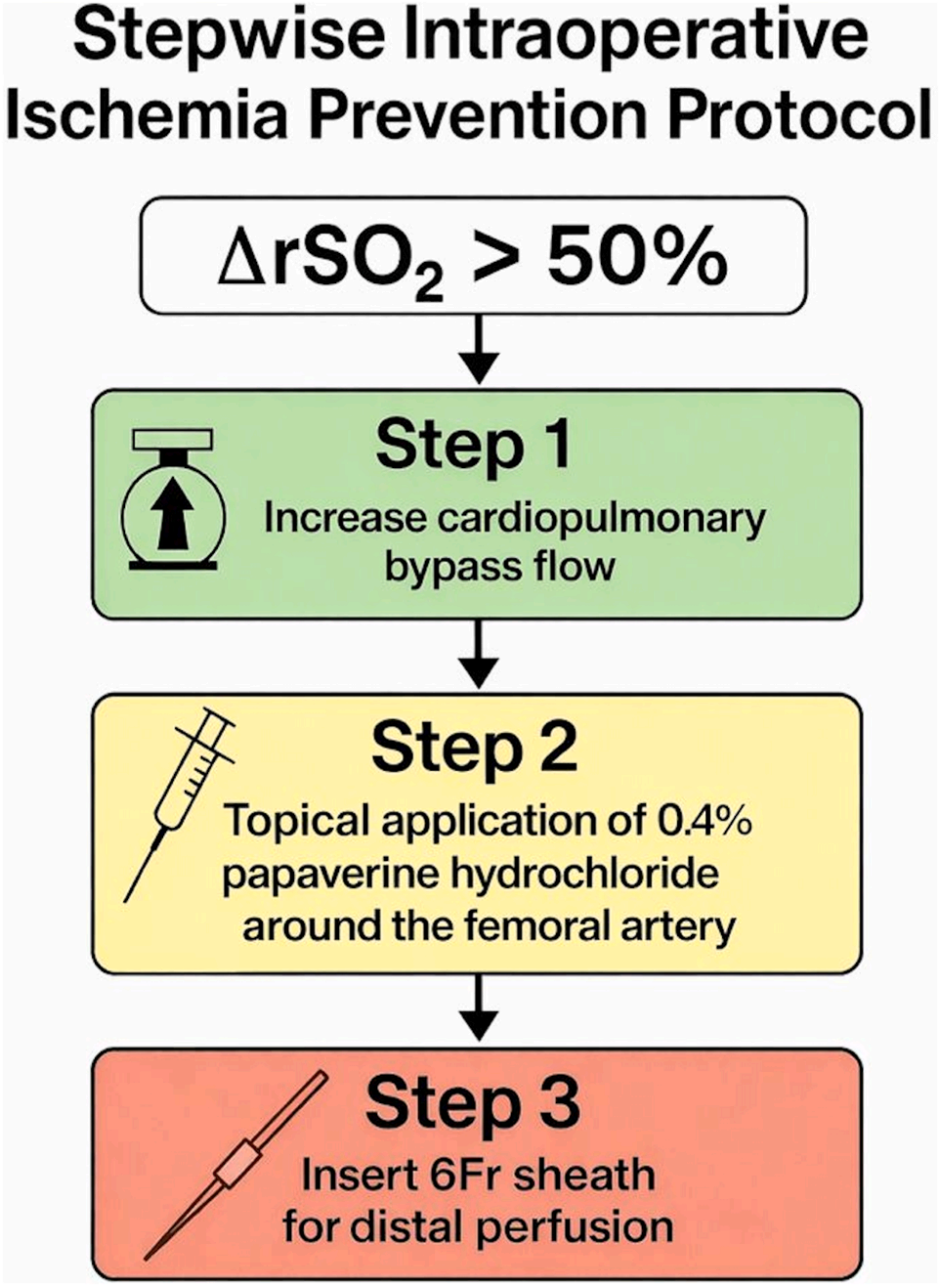
Stepwise intraoperative ischemia-prevention protocol. The protocol is triggered by a >50% decrease in regional oxygen saturation. Initial measures include increasing cardiopulmonary bypass flow and topical irrigation around the femoral artery with 0.4% papaverine hydrochloride solution. If regional oxygen saturation does not improve, distal perfusion is established using a 6-Fr sheath. *rSO*_*2*_, Regional oxygen saturation.

A >50% decrease in rSO_2_ (ΔrSO_2_ >50%) from baseline on the cannulated side was defined as significant and used to trigger our stepwise ischemia-prevention protocol. This definition was determined on the basis of previous institutional experience and intended to identify clinically meaningful reductions in limb perfusion. The protocol consisted of 3 measures: (1) increase CPB flow (depending on arterial pressure and venous drainage status, systemic flow was increased up to 2.7 L/min/m^2^); (2) topical irrigation around the FA with 5-10 mL of 0.4% papaverine hydrochloride solution (not injected intravascularly); and (3) insertion of a 6-Fr sheath into the distal FA to establish DP if rSO_2_ had not improved with the previous steps (Figure 2).

### End Points and Analysis

The primary end points were clinical limb ischemia, defined as the need for intraoperative DP or the development of postoperative compartment syndrome, and a ΔrSO_2_ >50%. The secondary end points included postoperative peak creatine kinase (CK) and lactate levels, 30-day mortality, stroke, and cannulation-related complications other than limb ischemia, defined as Clavien-Dindo grade III or greater. In addition, this study investigated correlations between rSO_2_ decrease and postoperative peak CK or lactate levels, as well as risk factors associated with intraoperative rSO_2_ decrease.

Clinical data were retrospectively collected from medical records. Continuous variables are presented as mean ± standard deviation. Statistical analyses were performed using JMP, version 17.0 (SAS Institute). Group comparisons were made using the Mann-Whitney *U* test. Univariate analyses of potential risk factors for rSO_2_ decline were performed using the Mann-Whitney *U* test and Fisher exact test, as appropriate. Variables with a *P* value < .1 in univariate analysis were included in the multivariate logistic regression model.

Candidate variables were required to be physiologically plausible risk factors for impaired limb perfusion, and the number of variables included in the multivariate analysis was limited according to the events-per-variable principle, with 1 variable per 10 outcome events. European System for Cardiac Operative Risk Evaluation (EuroSCORE) II a composite index incorporating ejection fraction, was excluded from the multivariate model to avoid overadjustment. To explore potential nonlinear associations, restricted cubic spline analyses were performed.

### Ethics

This study was approved by the institutional review board of Toranomon Hospital (approval no. 2385, approved on December 27, 2022), and written informed consent for the use of clinical data for research purposes was obtained from all patients. No external funding or specific grants were received for this study.

## Results

### Patient Characteristics

Preoperative patient characteristics are presented in Table 1. The mean age of the patients was 61.6 years, and 61.3% were male. On the cannulation side, the mean ankle-brachial index was 1.1, and the mean FA diameter was 9.5 mm.

**Table 1:** Patients’ characteristics

Variables	Mean ± SD/n (%)
Age, y	61.6 ± 13.7
Male sex	310 (61.3)
BSA, m^2^	1.6 ± 0.2
Reoperation	26 (5.1)
Hb, g/dL	13.4 ± 1.6
Cannulation site ABI	1.1 ± 0.1
FA diameter, mm	9.5 ± 1.7
EF, %	61.1 ± 8.0
EuroSCORE II, %	1.4 ± 1.5

*SD*, Standard deviation; *BSA*, body surface area; *Hb*, hemoglobin; *ABI*, ankle brachial index; *FA*, femoral artery; *EF*, ejection fraction; *EuroSCORE*, European System for Cardiac Operative Risk Evaluation.

### Operative Data

The surgical procedures consisted of mitral valve surgery in 47.1% of cases, aortic valve surgery in 25.3%, tricuspid valve surgery in 5.1%, and combined multiple valve procedures in 19.8%; nonvalvular surgery accounted for 2.6% of cases (Table 2). Concomitant atrial fibrillation ablation was performed in 10.9% of patients. The mean operation time was 195.1 minutes, the mean CPB time was 126.1 minutes, and the mean aortic crossclamp time was 88.7 minutes. Right-sided FA cannulation was performed in 98% of cases. The mean cannula size was 18.4 Fr, and the mean Cd/Fad was 66.5%. A cannula one size smaller than that recommended by body surface area was used in 52 of 505 patients (10.3%), according to the Cd/Fad criteria.

**Table 2:** Operative data

Variables	Mean ± SD/n (%)
Surgical procedure	
Aortic valve surgery	128 (25.3)
Mitral valve surgery	238 (47.1)
Tricuspid valve surgery	26 (5.1)
Double valve surgery	100 (19.8)
Nonvalvular surgery	13 (2.6)
Concomitant AF ablation surgery	55 (10.9)
Operation time, min	195.1 ± 47.0
CPB time, min	126.1 ± 37.4
ACC time, min	88.7 ± 30.7
Transfusion	113 (22.3)
FA cannulation site	
Right	499 (98.8)
Left	6 (1.2)
Cannula size, Fr	18.4 ± 2.0
Cd/Fad, %	66.5 ± 17.0

*SD*, Standard deviation; *AF*, atrial fibrillation; *CPB*, cardiopulmonary bypass; *ACC*, aortic crossclamp; *FA*, femoral artery; *Cd/Fad*, cannula-to-femoral artery diameter ratio.

### Clinical Limb Ischemia

No patient developed clinical limb ischemia, defined as the need for DP or postoperative compartment syndrome.

### rSO_2_ Decrease

A ΔrSO_2_ >50% was observed in 28 patients (5.5%). In all of these cases, the ischemia-prevention protocol was activated, and rSO_2_ promptly improved after increased CPB flow and topical irrigation around the FA with 0.4% papaverine hydrochloride solution. The median time from pump-on to the onset of ΔrSO_2_ >50% was 20.5 minutes, and the median recovery time from protocol activation to rSO_2_ normalization was 5.0 minutes.

### Risk Factors for rSO_2_ Decrease

Univariate analysis identified smaller FA diameter (odds ratio [OR], 0.72; 95% CI, 0.56-0.92; *P* = .01), lower ejection fraction (EF) (OR, 0.94; 95% CI, 0.91-0.98; *P* = .01), and greater EuroSCORE II (OR, 1.21; 95% CI, 1.01-1.42; *P* = .01) as significant risk factors for an intraoperative ΔrSO_2_ >50% (Table E2). Because the EuroSCORE II is a composite index incorporating EF, it was excluded from the multivariate analysis. A multivariate logistic regression analysis was performed using FA diameter and EF, both of which had a univariate *P* value < .1 and were physiologically plausible. In this model, both FA diameter (OR, 0.73; 95% CI, 0.56-0.94; *P* = .01) and EF (OR, 0.94; 95% CI, 0.91-0.98, *P* = .01) remained independently associated with a significant decrease in rSO_2_ (Table E3).

**Figure 3:**
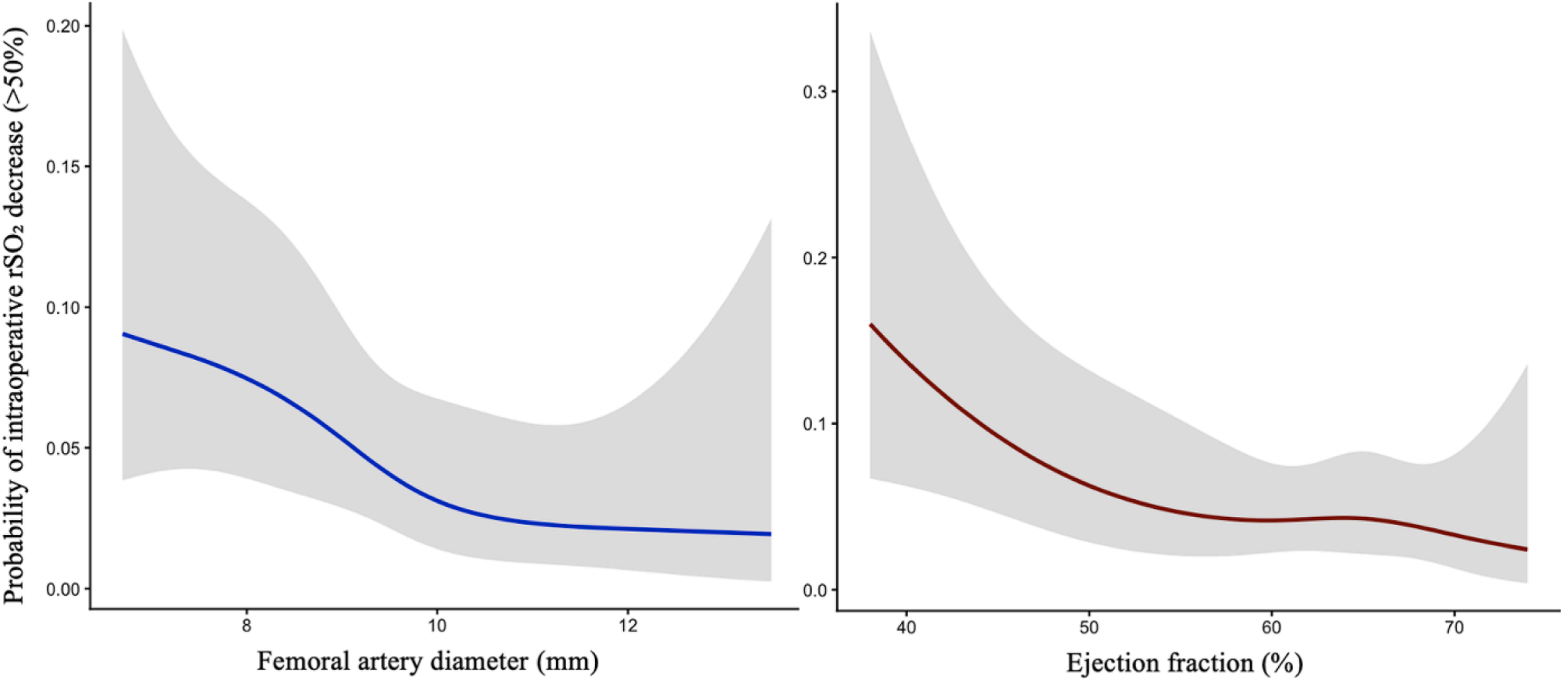
Restricted cubic spline analysis of femoral artery diameter and ejection fraction with the probability of intraoperative >50% decrease in regional oxygen saturation Both femoral artery diameter and ejection fraction were inversely associated with the probability of a >50% decrease in regional oxygen saturation in a nonlinear manner.

Restricted cubic spline analyses were performed for FA diameter and EF. The association between FA diameter and intraoperative ΔrSO_2_ >50% was more pronounced in smaller-diameter ranges, whereas the probability of rSO_2_ decrease plateaued at larger diameters. Similarly, lower EF was associated with a greater probability of rSO_2_ decrease, whereas this association appeared attenuated at greater EF levels (Figure 3).

### Association Between rSO_2_ Decrease and Peak CK/Lactate Levels

There were no significant differences in peak postoperative CK or lactate levels between patients with and without an intraoperative ΔrSO_2_ >50% (Table 3). Similarly, the frequency of concomitant ablation procedures, including pulmonary vein isolation and maze surgery, did not differ significantly between the 2 groups.

**Table 3:** Lactate and CK levels in cases with rSO_2_ decrease

Variables	rSO_2_ non-decrease (n = 477)	rSO_2_ decrease (n = 28)	*P* value
Peak CK, U/L	916.0 ± 600.8	872.4 ± 425.8	.79
Peak lactate, mmol/L	2.4 ± 1.3	2.7 ± 2.1	.70
Concomitant AF ablation surgery	52 (10.9)	3 (10.7)	1.00

Data are presented as mean ± SD or n (%). *CK*, Creatine kinase; *rSO*_*2*_, regional oxygen saturation; *AF*, atrial fibrillation; *SD*, standard deviation.

### 30-Day Mortality, Stroke, and Cannulation-Related Complications

No deaths occurred within 30 days after surgery (Table 4). Stroke was observed in 5 patients (1.0%). Cannulation-related complications were rare. No cases of intraoperative cannula dislodgement were observed. One patient developed a retroperitoneal hematoma caused by external iliac artery injury, which was treated with endovascular repair.

**Table 4:** Thirty-day mortality, stroke, and cannulation-related complications

Variables	n (%)
Mortality	0 (0)
Stroke	5 (1.0)
Aortic dissection	0 (0)
Arterial bleeding	1 (0.2)
FA pseudoaneurysm	0 (0)
Inguinal lymphatic leakage	4 (0.8)

Cannulation-related complications were defined as Clavien-Dindo grade III or greater, requiring surgical or interventional treatment. *FA*, Femoral artery.

## Discussion

This study demonstrated the clinical effectiveness of a standardized patient-selection strategy combined with a FA-cannulation technique and a structured ischemia-prevention protocol in minimizing limb ischemia during MICS. To our knowledge, this is the first report to provide a detailed description of a standardized FA-cannulation approach together with explicit selection criteria and to evaluate its clinical utility using intraoperative regional oxygen saturation monitoring. A key aspect of the present strategy was strict patient selection on the basis of preoperative vascular assessment. In this study, FA cannulation was avoided in patients with small-caliber arteries or significant atherosclerotic burden, as determined by CT. This selection process likely played an important role in minimizing distal limb ischemia and provided a foundation on which the standardized cannulation technique and ischemia-prevention protocol could be safely applied. In addition, exclusion of patients with advanced atherosclerotic disease may have contributed to reducing the risk of embolic complications, including perioperative stroke, by avoiding retrograde perfusion through diseased arterial segments.

Previous studies have proposed practical indications and contraindications for FA cannulation. Specifically, patients with peripheral arterial disease are considered unsuitable for FA cannulation^1^; FA cannulation is indicated when the FA diameter is >5 mm and the diameter difference between the FA and the cannula is >0.5 mm^2^; and patients showing on preoperative CT a thrombus thickness >3 mm, thrombus involving more than one third of the arterial circumference, or circumferential calcification in the aorta or iliac arteries are regarded as contraindicated for FA cannulation.^4^ On the basis of these findings, we applied a strict preoperative screening protocol, as described in the Methods, to all cases. Under this framework, unilateral FA perfusion was used in 505 of 576 consecutive MICS cases (87.7%) at our institution, whereas previous studies reported rates of 57.3%^1^ and 64.8%.^4^ Because unilateral FA perfusion remains the most common perfusion strategy in MICS, maintaining a high use rate while demonstrating both safety and efficacy is clinically meaningful.

Our FA-cannulation technique was originally described by Nagashima and colleagues^5^ at our center. It is characterized by 2 key features: (1) insertion of the arterial cannula at the minimum necessary depth and (2) dorsal orientation of the most proximal side hole. This orientation allows the side hole to face the distal FA and facilitates effective antegrade limb perfusion (Figure 1, *C*). To ensure reproducibility, we placed a 3-0 silk marker on the planned cannulation line before cannulation. Although shallow insertion may theoretically raise concerns regarding cannula dislodgement, no such events were observed in our cohort. This outcome is likely attributable to the application of a 3-point fixation method, which ensured secure stabilization of the cannula throughout the procedures.

In addition, we placed the purse-string suture longitudinally along the FA, a detail not explicitly described in the original report. This approach reduces luminal narrowing at the cannulation site after decannulation and may contribute to maintaining arterial patency.

To prevent limb ischemia in patients with smaller FAs, some institutions have adopted alternative strategies such as side-graft cannulation or bilateral FA cannulation.^6^ A side graft requires arterial anastomosis, which not only prolongs the time needed to establish CPB and increases the risk of vessel injury from clamping but also leaves a prosthetic graft remnant postoperatively that may serve as a potential source of infection, or late vascular or nerve complications. Bilateral FA cannulation similarly increases procedural complexity and the risk of vascular injury. At our institution, bilateral FA cannulation is considered one of the available options for arterial perfusion. However, when axillary artery perfusion is feasible, it is preferentially selected over bilateral FA cannulation. As a result, no patients in the present series underwent bilateral FA cannulation.

NIRS is widely used to monitor rSO_2_ in the lower limbs during MICS; however, there is no consensus on how intraoperative rSO_2_ changes should be interpreted or translated into clinical decision-making. As a result, reported incidences of significant rSO_2_ decline vary widely among institutions, ranging from 4.3% to 27.3%.^1^, ^2^, ^3^

In our cohort, a ΔrSO_2_ >50% was observed in only 5.5% of patients, which was comparable with or lower than the incidence reported in previous studies. Notably, no patients in our study required DP. FA cannulation is known to be associated not only with lower-limb ischemia and compartment syndrome, but also with a variety of vascular complications, including vessel perforation with hemorrhage, arterial dissection, cannula malposition, and pseudoaneurysm formation at the insertion site. DP, however, is not a perfect solution and carries procedure-specific risks, such as catheter occlusion and distal limb ischemia related to the small catheter diameter, as well as the potential for compartment syndrome caused by distal limb hyperperfusion due to limited control of perfusion flow.^7^ In the absence of standardized rSO_2_-based management strategies, some institutions initiate DP when ΔrSO_2_ exceeds 40% for a sustained period,^1^ whereas others adopt prophylactic approaches based on the Cd/Fad, with reported cutoff values ranging from 0.65 to 0.8.^2^^,^^8^^,^^9^

In contrast, our protocol defined a ΔrSO_2_ >50% as the activation threshold and implemented a stepwise management strategy, beginning with increased cardiopulmonary bypass flow, followed by topical application of 0.4% papaverine hydrochloride, and reserving DP only for cases refractory to these measures. With this approach, rSO_2_ improved in all activated cases through noninvasive interventions alone, and no instances of DP or postoperative compartment syndrome occurred. The median recovery time from protocol activation to rSO_2_ normalization was 5 minutes, indicating a rapid physiological response.

Smaller FA diameter and lower EF were suggested to be potential risk factors for intraoperative rSO_2_ decrease. This may reflect flow limitation in small-caliber vessels and reduced peripheral perfusion reserve. Given the limited number of events, this analysis should be interpreted as exploratory.

To assess whether transient rSO_2_ decline reflected clinically relevant ischemia, we examined postoperative biomarkers. Among patients who experienced a ΔrSO_2_ >50% and underwent protocol activation, postoperative CK and lactate levels did not differ significantly from those in patients without rSO_2_ decline, suggesting the absence of occult ischemia or enhanced anaerobic metabolism.

Previous studies have reported inconsistent associations between intraoperative rSO_2_ changes and postoperative biomarkers. Shikata and colleagues1^1^ demonstrated a positive correlation between the rate of rSO_2_ decline and postoperative CK and lactate levels, whereas Tarui and colleagues2^2^ found no association between the lowest rSO_2_ value and CK levels. These discrepancies suggest that the relative rate of rSO_2_ change, rather than its absolute value, may be more clinically relevant.

In our study, although a dichotomized threshold of ΔrSO_2_ >50% was used for protocol activation, no significant association was observed between rSO_2_ decline and postoperative biomarkers. This finding supports the validity of our stepwise protocol. Furthermore, despite the inclusion of patients undergoing concomitant ablation procedures such as pulmonary vein isolation or maze surgery, postoperative CK elevation remained modest compared with previous reports,^2^ further corroborating the overall safety of our approach.

Percutaneous FA cannulation has recently been reported and may be associated with lower rates of wound complications and lymphatic leakage.^10^ However, certain components of our standardized technique, such as precise control of cannula depth, directional orientation of the proximal side hole, and topical application of vasodilators, may be more difficult to achieve consistently with a percutaneous approach. In addition, the low incidence of wound complications and lymphatic leakage observed in our cohort may be attributable, at least in part, to our limited dissection strategy, which avoids circumferential vessel dissection and vessel taping.

The absence of clinical limb ischemia in more than 500 cases suggests that a standardized patient-selection strategy, combined with a standardized FA-cannulation technique and a stepwise ischemia-prevention protocol may provide a reproducible framework for safe FA perfusion in MICS. This integrated approach could serve as a practical reference for centers aiming to simplify perfusion management without compromising safety.

This study had several limitations. First, it was a single-center retrospective analysis; therefore, multicenter studies are warranted to validate the generalizability of our findings. Second, the study was conducted as a single-arm investigation without a control group, which limits direct comparison with alternative cannulation strategies. In the literature, detailed descriptions of FA-cannulation techniques are scarce and may vary among institutions. Prospective comparative studies are warranted to more objectively assess the relative effectiveness of our approach. Third, the cutoff value used to trigger our ischemia-prevention protocol (ΔrSO_2_ >50%) may not be universally optimal. Some centers employ a ΔrSO_2_ >40% threshold, and further studies are warranted to establish the most clinically appropriate cutoff value. However, in the present cohort, no adverse events were observed in association with interventions initiated using the ΔrSO_2_ >50% threshold, suggesting that a protocol based on earlier intervention may be safe.

## Conclusions

Using a standardized patient-selection strategy combined with a FA-cannulation technique and a structured ischemia-prevention protocol, we achieved safe unilateral femoral perfusion without major limb complications in 87.7% of MICS cases. None of the patients required DP or developed postoperative compartment syndrome, and intraoperative rSO_2_ reductions were comparable to or lower than those reported in previous studies. These findings suggest that this integrated, standardized approach provides a simple, reproducible, and clinically effective framework for safe femoral perfusion in MICS.

## Conflict of Interest Statement

The authors reported no conflicts of interest.

The *Journal* policy requires editors and reviewers to disclose conflicts of interest and to decline handling or reviewing manuscripts for which they may have a conflict of interest. The editors and reviewers of this article have no conflicts of interest.

## Figures and Tables

**Table E1:** Cannula size based on BSA

BSA, m^2^	<1.4	1.4-1.6	1.6-1.8	>1.8
Cannula size, Fr	15	17	19	21

*BSA*, Body surface area.

**Table E2:** Risk factors for rSO_2_ decrease (univariate analysis)

Variable	OR	95% CI	*P* value
Age	1.00	0.97-1.03	.92
Male sex	1.14	0.52-2.61	.74
BSA	0.22	0.03-1.36	.10
Reoperation	1.45	0.22-5.26	.64
Hb	0.93	0.73-1.18	.56
Canulation site ABI	0.17	0.01-3.55	.26
FA diameter	0.72	0.56-0.92	.01
EF	0.94	0.91-0.98	.01
EuroSCORE II	1.21	1.01-1.42	.01
Operation time	1.00	0.99-1.01	.39
CPB time	1.00	0.99-1.01	.30
ACC time	1.00	0.98-1.01	.82
Transfusion	1.69	0.74-3.86	.22
Cannulation site (right)	0.28	0.04-5.57	.32
Cannula size	0.90	0.75-1.08	.27
Cd/Fad	1.00	0.98-1.01	.42

*rSO*_*2*_, Regional oxygen saturation; *OR*, odds ratio; *BSA*, body surface area; *Hb*, hemoglobin; *ABI*, ankle brachial index; *FA*, femoral artery; *EF*, ejection fraction; *EuroSCORE II*, European System for Cardiac Operative Risk Evaluation II; *CPB*, cardiopulmonary bypass; *ACC*, aortic crossclamp; *Cd/Fad*, cannula-to-femoral artery diameter ratio.

**Table E3:** Risk factors for rSO_2_ decrease (multivariate analysis)

Variable	OR	95% CI	*P* value
FA diameter	0.73	0.56-0.94	.01
EF	0.94	0.91-0.98	.01

*rSO*_*2*_, Regional oxygen saturation; *OR*, odds ratio; *FA*, femoral artery; *EF*, ejection fraction.
